# Lysophosphatidic Acid Modulates TGF-β2-Induced Biological Phenotype in Human Conjunctival Fibroblasts

**DOI:** 10.3390/life14060770

**Published:** 2024-06-17

**Authors:** Megumi Watanabe, Yuri Tsugeno, Tatsuya Sato, Megumi Higashide, Nami Nishikiori, Araya Umetsu, Toshifumi Ogawa, Masato Furuhashi, Hiroshi Ohguro

**Affiliations:** 1 Departments of Ophthalmology, School of Medicine, Sapporo Medical University, S1W17, Chuo-ku, Sapporo 060-8556, Japan; watanabe@sapmed.ac.jp (M.W.); yuri.tsugeno@gmail.com (Y.T.); megumi.h@sapmed.ac.jp (M.H.); nami076@yahoo.co.jp (N.N.); araya.umetsu@sapmed.ac.jp (A.U.); 2 Departments of Cardiovascular, Renal and Metabolic Medicine, Sapporo Medical University, S1W17, Chuo-ku, Sapporo 060-8556, Japan; satatsu.bear@gmail.com (T.S.); a08m024@yahoo.co.jp (T.O.); furuhasi@sapmed.ac.jp (M.F.); 3 Departments of Cellular Physiology and Signal Transduction, Sapporo Medical University, S1W17, Chuo-ku, Sapporo 060-8556, Japan

**Keywords:** TGFβ2, human conjunctival fibroblast, 3D culture, lysophosphatidic acid

## Abstract

Background: Although lysophosphatidic acid (LPA) is known to have multiple pathophysiological roles, its contributions to ocular tissues, especially conjunctival fibrogenesis, remain to be elucidated. Methods: To study this issue, the effects of LPA on transforming growth factor-β2 (TGF-β2)-induced fibrogenesis of two-dimensional (2D) and three-dimensional (3D) cultures of human conjunctival fibroblasts (HconF) were examined by the following analyses: (1) planar proliferation determined by transepithelial electrical resistance (TEER) and fluorescein isothiocyanate (FITC)-dextran permeability measurements, (2) real-time metabolic analyses, (3) measurements of the size and stiffness of 3D spheroids, and (4) mRNA expression of extracellular matrix (ECM) molecules and their modulators. Results: LPA had no effect on TGF-β2-induced increase in the planar proliferation of HconF cells. LPA induced a more quiescent metabolic state in 2D HconF cells, but this metabolic suppression by LPA was partially blunted in the presence of TGF-β2. In contrast, LPA caused a substantial decrease in the hardness of 3D HconF spheroids independently of TGF-β2. In agreement with these different LPA-induced effects between 2D and 3D cultured HconF cells, mRNA expressions of ECM and their modulators were differently modulated. Conclusion: The findings that LPA induced the inhibition of both TGF-β2-related and -unrelated subepithelial proliferation of HconF cells may be clinically applicable.

## 1. Introduction

Lysophosphatidic acid (LPA), a bioactive lysophospholipid, is present in plasma, biological fluids, and tissues [1,2,3] and is involved in the activity of several G protein-coupled receptors (GPCRs) that mediate biological roles such as cell proliferation [4,5], migration [6,7], and the release of cytokines [8,9,10]. As of this writing, at least six LPA receptors, LPA 1 through 6, have been identified in mammals, and thus, such LPA-induced biological activities are mediated through specific LPA receptors coupled with members of the heterotrimeric G-protein family, including Gs, Gi, Gq, and G12/13 [11,12,13,14,15,16]. Alternatively, in addition to its physiological roles, it is also known that LPA-mediated signaling contributes to several pathological states such as wound repair [17], chronic viral infections [18], autoimmune diseases [19,20], allergic inflammation [21,22], obesity [23], and malignancy [4].

In the human eye, to maintain the normal barrier functions of the ocular surface, in addition to the cornea, conjunctiva should also be kept in healthy conditions, and both planar and subepithelial conjunctival fibrosis that occur in response to conjunctival wound healing therefore need to be appropriately controlled [24,25,26,27,28,29,30,31,32]. Alternatively, wound repairing mechanisms in the conjunctiva are critical factors involved in the pathogenesis of various ocular diseases and in the outcomes of ocular surgical intervention for pterygium and glaucoma [24,25,26]. During those wound repairing processes to which various cell types contribute, fibroblasts have been shown to be critically involved by their trans-differentiation into myofibroblasts [33]. In fact, as compared to naive fibroblasts, myofibroblasts have potent effects on cell proliferation, contractibility, and the production of components of the extracellular matrix (ECM). If prolonged stimulation of the activity of myofibroblasts is induced, hard scar tissue is produced and may affect the post-operative outcome of the ocular surface and glaucoma filter surgery. This suggests that control of the activity of conjunctival fibroblasts is an important key factor for the maintenance of ocular surface conditions and good vision [29,31,32]. In glaucoma filter surgery, the disorganized increase of ECM secretion and its deposition in the subconjunctival environment is stimulated by cytokines, including transforming growth factor-β (TGF-β), tumor necrosis factor-α (TNF-α), vascular endothelial growth factor (VEGF), and interleukin-1 (IL-1) [30,34], which originate from the aqueous humor (AH), in addition to Tenon’s capsule and conjunctival fibroblasts [35,36]. In fact, a recent study by Gater et al. showed that proliferation of Tenon’s capsule fibroblasts and conjunctival tissue fibroblasts (TCCT) substantially and synergistically increased with TGF-β, TNF-α, VEGF, and AH exposure by using two-dimensional (2D) and three-dimensional (3D) TCCT models [37], suggesting that an in vitro 3D culture model is useful in addition to a conventional 2D culture model for studying subconjunctival fibrogenesis. Our group we independently developed suitable in vitro conjunctival 2D and 3D HconF models for fibrogenic changes on the plane or in the spatial space, respectively [38]. In contrast to the well-characterized factor TGF-β for subconjunctival fibrogenesis [33,39,40], as of this writing, our knowledge of LPA-mediated signaling within ocular pathophysiology, especially conjunctival fibrosis, remains quite limited. However, since previous studies have shown that (1) LPA is involved in the etiology of glaucoma [41,42,43] and corneal wound healing [44] and (2) the AH levels of both LPA and TGF-β2 are increased in patients with glaucoma [43,45], it would be of great interest to have a better understanding of the nature of the effects of both factors in conjunctival fibrosis.

Therefore, in the current study, to determine the nature of the contributions of LPA-mediated signaling within conjunctival fibrosis, those issues were investigated using our recently developed suitable in vitro 2D and 3D cultured HconF cell models [38]. 

## 2. Materials and Methods

To use commercially available human conjunctival fibroblasts (HconF) from ScienCell Research Laboratories (Carlsbad, CA, USA), we complied with the tenets of the Declaration of Helsinki, and all experimental protocols were approval by the internal review board (IRB) of Sapporo Medical University.

### 2.1. Preparation of the 2D and 3D Cultured HconF Cells

HconF cells were subjected to 2D planar culture in 2D culture dishes (150 mm in diameter) at 37 °C in the Fibroblast Medium (FM, Cat. #2301, ScienCell Research Laboratories, Carlsbad, CA, USA) [38]. They were maintained by changing the medium every other day or further processed into 3D spheroid cultures, as described previously [38]. Briefly, pellets of the 2D HconF cells prepared by washing with phosphate-buffered saline (PBS) and treatment with 0.25% Trypsin/EDTA were resuspended in Fibroblast Medium supplemented with 0.25% methylcellulose (Methocel A4M). A 28-μL cell suspension containing 20,000 HconF cells was seeded into each hanging drop culture plate (#HDP1385, Sigma-Aldrich, St. Louis, MO, USA). Subsequently, 3D spheroid cultures were maintained by changing half of the medium (14 μL) until Day 6. For testing drug-induced effects, 5 ng/mL TGF-β2 and/or 500 nM LPA were added to 2D or 3D cultured HconF cells daily from Day 1 to Day 6. The doses of TGF-β2 used in the current study were based on previous equivalent methods [38,46], and the doses of LPA was determined by a previous equivalent method using cultured rabbit corneal cells [47] and AH LPA levels of glaucoma patients [43]. 

### 2.2. Planar Proliferation Analysis of the HconF Cell Monolayer

Two-dimensional (2D) HconF monolayers on wells of the TEER plate (pore size: 0.4 μm, diameter: 12 mm, Corning Transwell, Sigma-Aldrich) were prepared as described above. On Day 6, TEER electrical resistance (Ωcm^2^) was measured by using an electrical probe equipped in the TEER measurement system (Kanto Chemical Co., Inc., Tokyo, Japan). Alternatively, measurement for FITC-dextran permeability was performed by detecting the fluorescence intensity that permeated through the membrane from the basal compartment to the apical compartment during a period of 60 min as described previously [48]. 

### 2.3. Real-Time Measurement for Cellular Metabolic Functions

Using a Seahorse XFe96 Bioanalyzer (Agilent Technologies, Santa Clara, CA, USA), mitochondrial function by the oxygen consumption rate (OCR) and glycolysis function by the extracellular acidification (ECAR) of 2D HconF cells were simultaneously measured. The analysis was performed by sequential administrations of oligomycin (2.0 μM), carbonyl cyanide p-trifluoromethoxyphenylhydrazone (FCCP, 5.0 μM), a mixture of rotenone (1.0 μM) and antimycin A (1.0 μM), and 2-deoxyglucose (2-DG, 10 mM). The proton efflux rate (PER) converted from ECAR was calculated using the rate of glycolytic ATP production by Seahorse XF Wave software version 2.6 (Agilent Technologies, Santa Clara, CA, USA), as described previously [49]. 

### 2.4. Evaluation of the Size and Hardness of HconF Cell 3D Spheroids

As physical aspects of the 3D HconF spheroids, their largeness and hardness were measured as described previously [50,51]. In brief, the mean largeness of the 3D spheroids was determined as the largest cross-sectional area of the phase contrast microscopy image obtained by using an inverted microscope (Nikon ECLIPSE TS2, Tokyo, Japan) using ImageJ software version 1.51n (National Institutes of Health, Bethesda, MD, USA). For the measurement of hardness of each living 3D spheroid, a single spheroid placed on a 3-mm × 3-mm plate was compressed until its diameter reached to its semi-diameter during a period of 20 s using a micro-compressor system (MicroSquisher, CellScale, Waterloo, ON, Canada). The requiring force/displacement (μN/μm) was used as an index for hardness of a 3D spheroid.

### 2.5. Immunocytochemistry of 3D HconF Spheroids

Immunocytochemistry of the 3D HconF cells was carried out as described previously [52,53]. Briefly, 4% paraformaldehyde-fixed 3D spheroids were incubated in 3% BSA in PBS for 3 h. After washing twice with PBS for 30 min each time, 3D spheroids were sequentially exposed to 1:200 dilution of primary antibodies, including an anti-human COL1 (#600-401-103-0.1, ROCKLAND Antibodies & Assays, Limerick, PA, USA), COL4 (#600-401-106S, ROCKLAND Antibodies & Assays, Limerick, PA, USA), COL6 (#009-001-108, ROCKLAND Antibodies & Assays, Limerick, PA, USA), and FN (#A-11, Santa Cruz Biotechnology, Inc., Dallas, TX, USA) rabbit antibody at 4 °C overnight. The 3D spheroids were then washed 3 times with PBS for 1 h each time and exposed to 1:1000 dilution of secondary antibodies, including a goat anti-rabbit IgG (488 nm, #A27034, Invitrogen, Waltham, MA, USA) with phalloidin (594 nm, 1:1000 dilution, #17466-45-4, Cayman Chemical, Ann Arbor, MI, USA) and DAPI (1:1000 dilution, #D523-10, DOJINDO, Osaka, Japan) for 3 h. Then, after mounting on ProLong Gold Antifade Mountant with a cover glass, immunofluorescent images were taken with a Nikon A1 confocal microscope using a ×20 air objective at a resolution of 1024 × 1024 pixels.

### 2.6. qPCR Analysis

As described previously [52,53], 2D and 3D cultured HconF cells were subjected to total RNA extraction (RNeasy mini kit, Qiagen, Valencia, CA, USA) and reverse-transcribed (SuperScript IV kit, Invitrogen) according to the instructions of the manufacturers. Quantification of each respective gene expression was performed by a StepOnePlus machine (Applied Biosystems/Thermo Fisher Scientific, Waltham, MA, USA) using Universal Taqman Master mix and specific primers and probes (Supplemental Table S1). Normalization of cDNA quantities was done by using the expression of 36B4 (Rplp0) as the control.

### 2.7. Statistical Analyses

All data are shown as arithmetic means ± SEM. Statistical analyses were carried out using Graph Pad Prism 8 (GraphPad Software, San Diego, CA, USA) as described previously [52,53]. A significant difference of less than 0.05 was determined by ANOVA followed by Tukey’s multiple comparison test or Student’s *t*-test.

## 3. Results

**Figure 1:**
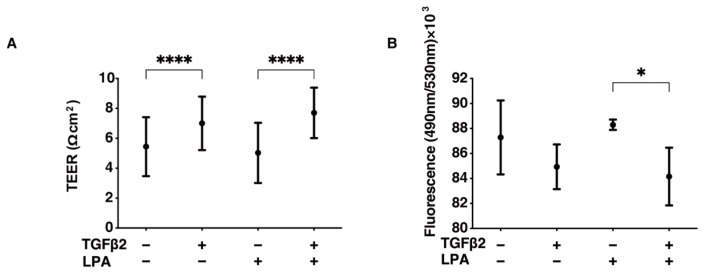
LPA-induced effects on planar proliferation of 2D HconF cells. For evaluation of the planar proliferation of the HconF monolayer, we measured the TEER electric resistance (Ωcm^2^) (panel (**A**)) and FITC-dextran permeability (panel (**B**)) of 2D HconF cells that had been incubated with 5 ng/mL TGF-β2 and/or 500 nM LPA, in addition to non-treated control cells. Measurements were conducted in triplicate using fresh preparations (*n* = 4 each). * *p* < 0.05 and **** *p* < 0.001 (ANOVA followed by Tukey’s multiple comparison test).

**Figure 2:**
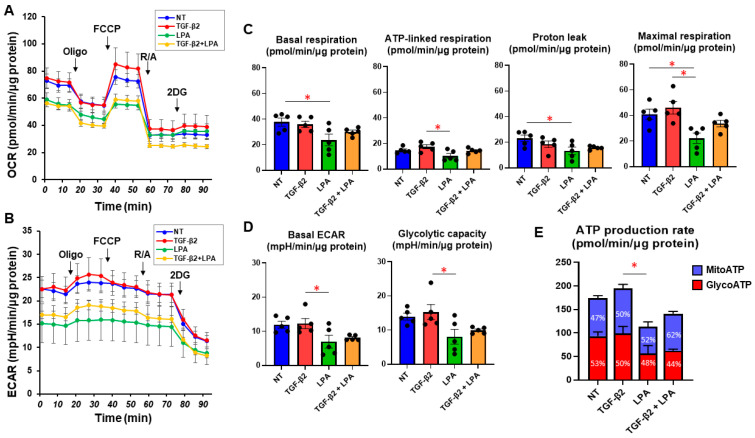
LPA-induced effects on cellular metabolic functions of 2D HconF cells. In 2D HconF cells that had been incubated with 5 ng/mL TGF-β2 and/or 500 nM LPA in addition to non-treated control cells (*n* = 6 each), real-time metabolic function was assessed by measuring the oxygen consumption rate (OCR, (**A**)) and extracellular acidification rate (ECAR, (**B**)) using a Seahorse XFe96 Bioanalyzer (*n* = 5 each). After measurements for the baseline, oligomycin (Oligo, complex V inhibitor), FCCP (a protonophore), rotenone/antimycin A (R/A, complex I/III inhibitors), and 2DG (a hexokinase inhibitor) were consecutively added. The key indices for mitochondrial function (**C**) and glycolysis (**D**) are shown. The proportions of MitoATP and GlycoATP production rates are shown in (**E**). * *p* < 0.05 (Student’s *t*-test).

To study the effects of LPA on conjunctival fibrogenesis induced by TGF-β2, we used 2D and 3D cultures of HconF cells that were established in vitro models replicating the TGF-β2-induced fibrogenic changes on the planar epithelial plane and spatial subepithelial space, respectively [38]. Prior to the current study, among the six known LPA receptors, the mRNA expression level of the LPA4 receptor was three times higher than the expression levels of the LPA1–3 receptors, but no expression of the LPA5 or -6 receptors was detected in the 2D and 3D HconF preparations (Figure S1) Followingly, in the absence or presence of 500 nM LPA, TGF-β2-induced planar proliferation was evaluated by measurements of TEER and FITC-dextran permeability of 2D HconF monolayers. The TEER values were substantially increased, and the FITC-dextran permeability was relatively decreased by the administration of TGF-β2, as shown in our previous studies [38,54,55] (Figure 1). However, although LPA had no significant effects on planar proliferation, as assessed by TEER and FITC-dextran permeability in the absence or presence of TGF-β2, the cellular metabolic state in 2D HconF cells was shifted to a more quiescent state by treatment with LPA, resulting in a reduced rate of ATP production (Figure 2). LPA appeared to partially blunt the effects of TGF-β2, but this effect was not statistically significant. These results suggest that LPA may suppress cellular metabolic functions in either the absence or presence of TGF-β2, despite the fact that no significant effects on planar proliferation of the 2D cultured HconF cells was observed. 

**Figure 3:**
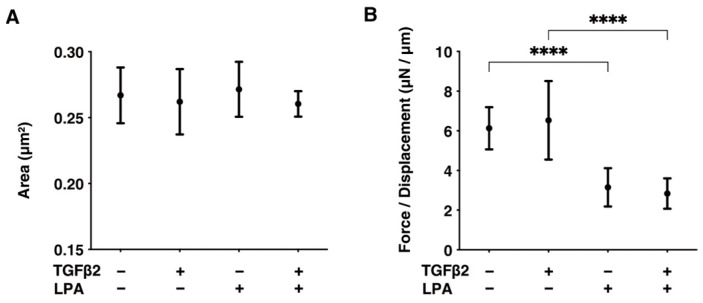
LPA-induced effects on the physical aspects, including largeness and hardness, of 3D HconF spheroids. The 3D HconF spheroids that had been incubated with 5 ng/mL TGF-β2 and/or 500 nM LPA in addition to non-treated control cells were subjected to measurements for their largeness (**A**) and hardness (**B**). Measurements were conducted in triplicate using fresh preparations (*n* = 16 each). **** *p* < 0.001 (ANOVA followed by Tukey’s multiple comparison test).

Next, to examine the effects of LPA on TGF-β2-induced fibrogenesis that occurs in the subepithelial spatial environment, the physical characteristics, including the largeness and hardness of the 3D spheroids of HconF cells, were evaluated. Although the mean areas of the 3D HconF spheroids were essentially unchanged by TGF-β2 and/or LPA, their hardness was substantially decreased by LPA, and this was independent of TGF-β2. These collective results showed that LPA markedly suppresses TGF-β2-induced fibrogenesis, as well as TGF-β2-unrelated proliferation, in subepithelial spatial environments (Figure 3). 

**Figure 4:**
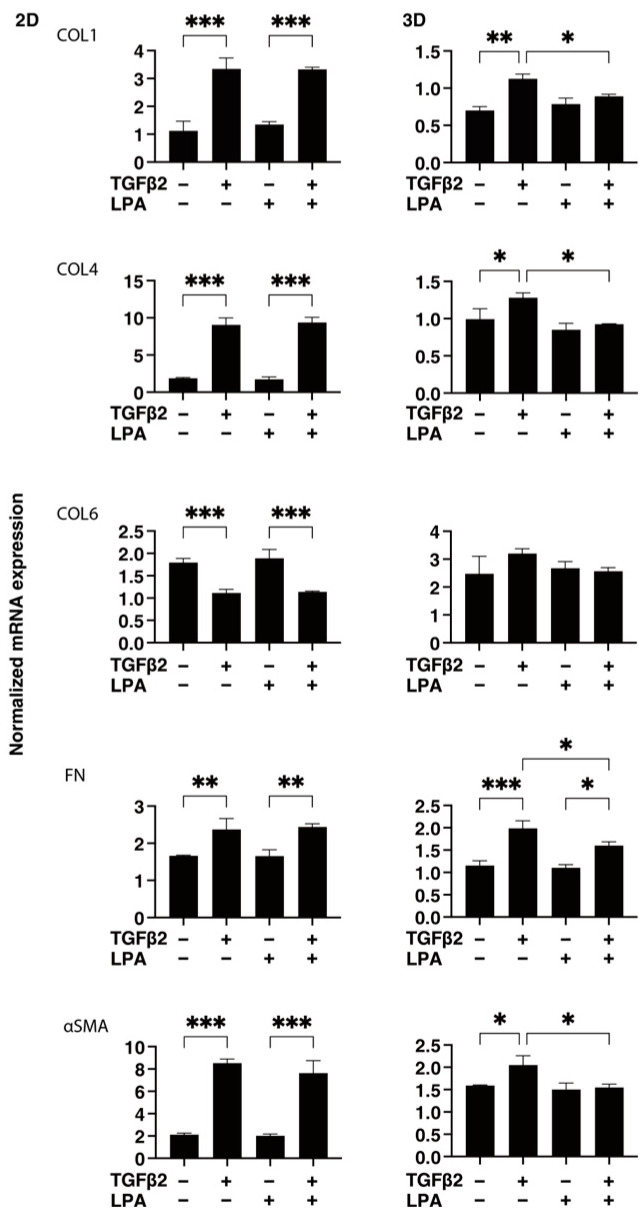
LPA-induced effects on gene expression of ECM proteins *COL1*, *COL4*, *COL6*, *FN*, and *αSMA* in 2D and 3D cultured HconF cells. The 2D and 3D HconF cells that had been incubated with 5 ng/mL TGF-β2 and/or 500 nM LPA in addition to non-treated control cells were subjected to qPCR analysis. Measurements were conducted in triplicate using fresh preparations (2D: *n* = 5; 3D: *n* = 10 spheroids each). * *p* < 0.05, ** *p* < 0.01, and *** *p* < 0.005 (ANOVA followed by Tukey’s multiple comparison test).

**Figure 5:**
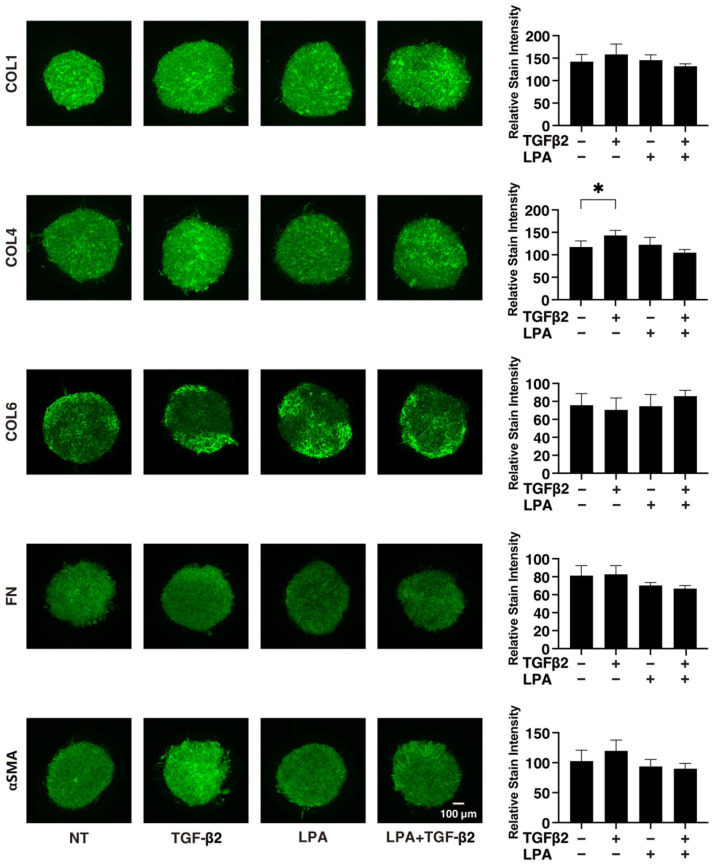
Immunolabeling of ECM proteins *COL1*, *COL4*, *COL6*, *FN*, and *αSMA* of the 3D HconF spheroids. The 3D HconF spheroids that had been incubated with 5 ng/mL TGF-β2 and/or 500 nM LPA in addition to non-treated control cells were subjected to immunocytochemistry analysis. Analyses were conducted in triplicate using fresh preparations (*n* = 5). Relative staining intensities of the molecules are plotted in the right panels. * *p* < 0.05 (ANOVA followed by Tukey’s multiple comparison test).

**Figure 6:**
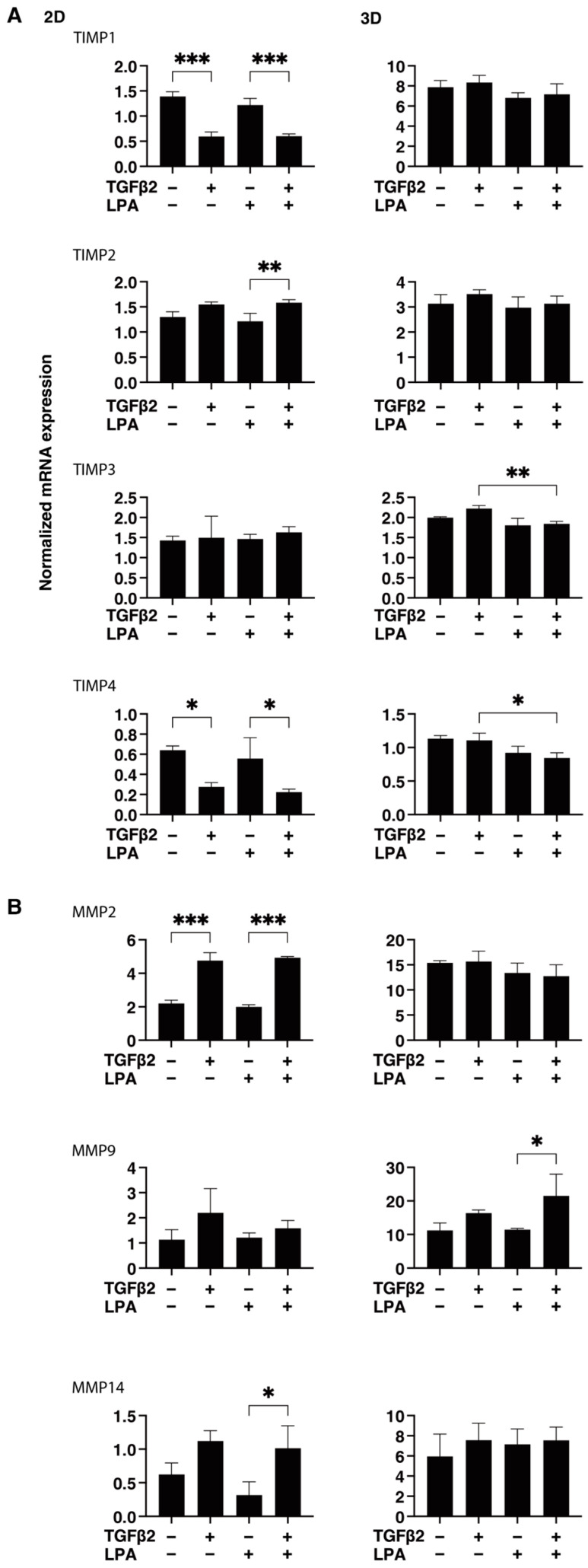
LPA-induced effects on gene expression of *TIMP1-4* (**A**) and *MMP2*, *-9*, and -*14* (**B**) in 2D and 3D cultured HconF cells. The 2D and 3D HconF cells that had been incubated with 5 ng/mL TGF-β2 and/or 500 nM LPA in addition to non-treated control cells were subjected to qPCR. Measurements were conducted in triplicate using fresh preparations (2D: *n* = 5; 3D: *n* = 10 spheroids each). * *p* < 0.05, ** *p* < 0.01, and *** *p* < 0.005 (ANOVA followed by Tukey’s multiple comparison test).

To gain further insights into the underlying mechanisms responsible for causing the above characteristic effects by LPA and/or TGF-β2 at the molecular level, the expression of ECM proteins, including collagen (COL)1, -4, and -6; fibronectin (FN); and α smooth muscle actin (αSMA), was evaluated by qPCR analysis and immunocytochemistry. As shown in Figure 4, significant TGF-β2-induced upregulation of all five ECM proteins, except for COL6, and the downregulation of COL6 were observed in the absence and presence of LPA in the 2D cultures. In contrast to the 2D cultured cells, the expression of all five ECM proteins, except for COL6, in 3D HconF spheroids was substantially upregulated by TGF-β2, and such TGF-β2-induced changes were markedly inhibited by LPA. In terms of the different mRNA expression levels of ECM proteins between the 2D and 3D cell cultures, it was speculated that the difference in cell–cell interactions may be related—that is, the 2D cell culture: side by side on the flat vs. 3D cell culture: omnidirectional cell–cell interaction in the spatial environment, as described recently [56]. In addition, the mRNA expression of each ECM protein in 3D HconF spheroids was similar to the immunolabeling level (Figure 5), but some differences were also observed between the analyses. In fact, these differences in the levels of gene expression between the 2D and 3D cell cultures, and in the level of expression in between gene expression and immunolabeling, were sometimes observed in our previous studies [54,55,57]. Consistent with these findings, in the 2D cells, the expression of TIMP 1 and -4 and the expression of MMP2 and -14 were downregulated and upregulated, respectively, by TGF-β2 but not by LPA. In contrast, although significant changes in the TIMPs and MMPs in the case of 3D spheroids were not induced by TGF-β2 and/or LPA, LPA significantly suppressed the expression of TIMP 3 and -4 in the presence of TGF-β2 (Figure 6). These collective findings suggest that, although LPA by itself did not cause either planar proliferation or subepithelial fibrogenic changes in HconF cells, LPA suppressed the TGF-β-induced fibrogenic changes in 3D HconF spheroids.

## 4. Discussion

LPA consists of a phosphate head group connected through a glycerol backbone to a fatty acyl chain with different lengths and saturation degrees and is found in various locations, such as membrane lipids [58,59]. LPA molecules serve as bioactive ligands that link through cognate receptors (LPAR1–6) to induce a variety of physiological functions, including cell growth, inflammation, and differentiation [60]. Such LPA-mediated signaling has also been shown to be involved in the pathophysiology associated with ocular tissues. For example, an elevated level of LAP is detected in injured cornea, suggesting that LPA is indeed involved in the proliferation and wound-healing process of cornea epithelia [61]. Furthermore, it was recently shown that elevated levels of LPA were detected in the AH in patients with glaucoma compared with the levels in non-glaucoma subjects, suggesting that LPA signaling may importantly be involved in the etiology of glaucoma [43,45]. Since mitomycin C-assisted glaucoma filtering surgeries are still the standard surgical procedures for treating cases of glaucoma [62], it would be interesting to study the effects of LPA on conjunctiva fibrosis in cases in which the AH is exuded into the subconjunctival filtering space after glaucoma filtering surgery. However, as far as we surveyed, no related study has been made available. In the current study, we investigated the LPA-induced effects on planar and subepithelial conjunctival fibrogenesis that could be replicated by our recently developed 2D and 3D culture methods using TGF-β2 [38,54,55], and the following results were obtained: (1) among the six known LPARs, LPAR1–4 were expressed in HconF cells, and LPAR4 was the most abundantly expressed, although no expression of LPAR5 or -6 was detected; (2) planar proliferation, as evaluated by measurements using TEER and FITC dextran permeability, was not affected by LPA, although LPA significantly modulated mitochondrial and glycolytic functions in both 2D HconF cells treated and not treated with TGF-β2; and (3) LPA substantially decreased the subepithelial fibrogenic changes, as evidenced by measurements of the hardness of TGF-β2-treated and -untreated 3D HconF spheroids. Based on these results, we conclude that LPA could inhibit both TGF-β2-related and -unrelated subepithelial proliferation of HconF cells, suggesting that AH LPA may exert protective effects on subconjunctival fibrosis in the case of glaucoma filtering surgery. However, based on the results of qPCR analyses of several ECM proteins and their modulators in 3D spheroids, LPA was not the sole cause of the subepithelial fibrogenic changes in HconF cells. Therefore, additional and currently unidentified underlying mechanisms may be involved in the LPA-induced decrease in the hardness of TGF-β2-untreated 3D HconF spheroids. In fact, it has been shown that LPA receptor signaling has been differently involved in a number of physiological and pathological functions [60]. Furthermore, although we have no explanation for why such a LPA-mediated effect was observed in the 3D cultures but not in the 2D cultures, we speculate that the 3D spheroids may have different biological aspects from those of the 2D cultured cells, even when the same cells are used. In fact, our recent study showed diverse biological differences between 2D and 3D cultured 3T3-L1 cells, and it is possible that such differences could be induced by STAT3, which was identified as the upstream master regulatory gene by RNA sequencing analysis [63]. If such diversity was also observed in HconF cells, our current observations would provide support for our previously proposed idea that 2D and 3D cultured HconF cells may be useful in studies of planar and subepithelial fibrogenic changes in the human conjunctiva [38]. 

To elucidate the possible effects of LPA on the surgical outcome in glaucoma filtering surgery, Igarashi et al. showed by multivariate analyses of 70 glaucomatous eyes in which trabeculectomy had been performed that the AH levels of autotaxin (ATX), an upstream regulator of LPA, were significantly correlated with the number of needling procedures during the past 12 post-operative months. That is, the surgical success rates for exfoliative glaucoma in which the ATX levels were significantly high may be lower due to the need for increased needling. In addition, fibrotic changes were enhanced by ATX treatment in 2D cultured HconF cells, and the fibrotic changes were significantly inhibited by an ATX inhibitor. These findings suggest that AH ATX and LPA may become important factors for estimating the fibrotic response in HconF cells and survival of the glaucoma filtering bleb formation [45]. However, in terms of subconjunctival fibrogenic changes after glaucoma filtering surgery, it has been suggested that various cytokines, chemokines, and other signaling molecules are involved, in addition to ATX-LPA signaling [64]. Furthermore, in their in vitro experiments, 2D cultured HconF cells were treated with extremely high doses of ATX (44 μM) compared to the physiological concentrations detected in specimens obtained from patients (0.5–0.8 nM) [45]. Therefore, their conclusions are most likely overstated, and the roles of LPA in subconjunctival fibrogenic changes after glaucoma surgery therefore need to be reevaluated, since the results reported by those researchers are contrary to the findings in our study. 

In the ATX-LPA-LPA receptor signaling axis, LPA moves to the surface of the specific target cell and binds to its LPAR1–6. Then, LPA stimulates specific LPAR downstream signal cascades related to phosphatidylinositide 3-kinase (PI3K), phospholipase C (PLC), Rho, mitogen-activated protein kinase (MAPK), or adenylate cyclase (AC) through different G proteins [65]. In the current study, we found that LPAR1–4 molecules were expressed in the 2D and 3D cultured HconF cells and that LPAR4 was the major component. However, as of this writing, the physiological and pathological significance of LPAR4 in conjunctival cells remains to be elucidated. Previous studies in both mice and zebrafish have suggested that LPAR4 plays a role in vascular development [66,67], vascular network formation by promoting cell–cell contact [68,69], the regulation of lymphocyte transmigration [70], and influencing hematopoiesis [71]. Considering these collective observations, the effects of LPA on the 3D HconF spheroids reported herein may also be related to cell–cell interactions within the spatial 3D spheroid architecture. 

However, this rationale remains speculative at present, and the roles of LPA signaling in biological activities in conjunctival cells have not yet been fully elucidated. Furthermore, as a limitation of this study, there have been controversial observations of LPA-induced effects on cell proliferation and fibrogenesis of ocular tissues. For instance, it has been reported that LPA stimulates proliferation of the three major cell types of the cornea (epithelial cells, keratocytes, and endothelial cells) and that this stimulation is dose-dependent [44,72]. Furthermore, autotaxin (ATX) and LPA signaling have been suggested to be involved in the pathogenesis of glaucoma by increase of the AH outflow facility [41,45]. However, our 3D HconF spheroids were prepared by using a drop culture method in which no scaffolds are required, unlike in other preparation methods [56]. Thus, this drop culture method was the simplest method and allowed us to prepare extremely reproducible 3D spheroids of non-cancerous cells, including 3T3-L1 cells [52], human orbital fibroblasts [50], human trabecular meshwork cells [73], human scleral stromal fibroblast [74], and human corneal stromal fibroblasts [75], as well as cancerous cells, including malignant melanoma [76], A549 cells [49], and oral squamous cell carcinoma cells [77]. Furthermore, our method for measurements of the hardness of 3D spheroids is not only easily reproducible and reliable but also the only method for evaluating the physical hardness of living 3D spheroids. Collectively, we assumed that our results using this method may closely reflect the in vivo nature of conjunctival fibrogenesis. In support of this, it has been shown that ATX and LPA signaling mechanisms are also required for normal wound healing, in addition to pathogenic conditions [41]. Therefore, additional investigations, including knockdown/knockout assays and the use of pharmacological inhibitors of LPA receptors and others, will be required to confirm this conclusion, which is our next project.

## 5. Conclusions

Using HconF cells and the 2D and 3D cell culture methods, we evaluated LPA-induced effects on conjunctival fibrogenesis and found that LPA inhibited both the TGF-β2-related and -unrelated subepithelial proliferation of HconF cells, and thus, this may be useful information in clinical practice.

## Data Availability

The data that support the findings of this study are available from the corresponding author upon reasonable request.

## Supplementary Materials

The following supporting information can be downloaded at https://www.mdpi.com/article/10.3390/life14060770/s1: Figure S1: The mRNA expression of LPAR1–6 of 2D cultured HconF cells. Figure S2: Immunolabeling of ECMs of the 2D Cultured HconF cells. Table S1: The sequences of the primers and probes used in this study.

## References

[1] Aoki J., Taira A., Takanezawa Y., Kishi Y., Hama K., Kishimoto T., Mizuno K., Saku K., Taguchi R., Arai H. (2002). Serum lysophosphatidic acid is produced through diverse phospholipase pathways. J. Biol. Chem..

[2] Postma F.R., Jalink K., Hengeveld T., Bot A.G., Alblas J., de Jonge H.R., Moolenaar W.H. (1996). Serum-induced membrane depolarization in quiescent fibroblasts: Activation of a chloride conductance through the G protein-coupled LPA receptor. Embo J..

[3] Tigyi G., Miledi R. (1992). Lysophosphatidates bound to serum albumin activate membrane currents in Xenopus oocytes and neurite retraction in PC12 pheochromocytoma cells. J. Biol. Chem..

[4] Mills G.B., Moolenaar W.H. (2003). The emerging role of lysophosphatidic acid in cancer. Nat. Rev. Cancer.

[5] van Corven E.J., Groenink A., Jalink K., Eichholtz T., Moolenaar W.H. (1989). Lysophosphatidate-induced cell proliferation: Identification and dissection of signaling pathways mediated by G proteins. Cell.

[6] Hao F., Tan M., Xu X., Han J., Miller D.D., Tigyi G., Cui M.Z. (2007). Lysophosphatidic acid induces prostate cancer PC3 cell migration via activation of LPA(1), p42 and p38alpha. Biochim. Biophys. Acta.

[7] van Leeuwen F.N., Giepmans B.N., van Meeteren L.A., Moolenaar W.H. (2003). Lysophosphatidic acid: Mitogen and motility factor. Biochem. Soc. Trans..

[8] Zhao Y., He D., Saatian B., Watkins T., Spannhake E.W., Pyne N.J., Natarajan V. (2006). Regulation of lysophosphatidic acid-induced epidermal growth factor receptor transactivation and interleukin-8 secretion in human bronchial epithelial cells by protein kinase Cdelta, Lyn kinase, and matrix metalloproteinases. J. Biol. Chem..

[9] Zhao Y., He D., Zhao J., Wang L., Leff A.R., Spannhake E.W., Georas S., Natarajan V. (2007). Lysophosphatidic acid induces interleukin-13 (IL-13) receptor alpha2 expression and inhibits IL-13 signaling in primary human bronchial epithelial cells. J. Biol. Chem..

[10] Zhao Y., Usatyuk P.V., Cummings R., Saatian B., He D., Watkins T., Morris A., Spannhake E.W., Brindley D.N., Natarajan V. (2005). Lipid phosphate phosphatase-1 regulates lysophosphatidic acid-induced calcium release, NF-kappaB activation and interleukin-8 secretion in human bronchial epithelial cells. Biochem. J..

[11] An S., Dickens M.A., Bleu T., Hallmark O.G., Goetzl E.J. (1997). Molecular cloning of the human Edg2 protein and its identification as a functional cellular receptor for lysophosphatidic acid. Biochem. Biophys. Res. Commun..

[12] Chun J. (1999). Lysophospholipid receptors: Implications for neural signaling. Crit. Rev. Neurobiol..

[13] Fukushima N., Kimura Y., Chun J. (1998). A single receptor encoded by vzg-1/lpA1/edg-2 couples to G proteins and mediates multiple cellular responses to lysophosphatidic acid. Proc. Natl. Acad. Sci. USA.

[14] Im D.S., Heise C.E., Harding M.A., George S.R., O’Dowd B.F., Theodorescu D., Lynch K.R. (2000). Molecular cloning and characterization of a lysophosphatidic acid receptor, Edg-7, expressed in prostate. Mol. Pharmacol..

[15] Lee C.W., Rivera R., Gardell S., Dubin A.E., Chun J. (2006). GPR92 as a new G12/13- and Gq-coupled lysophosphatidic acid receptor that increases cAMP, LPA5. J. Biol. Chem..

[16] Noguchi K., Ishii S., Shimizu T. (2003). Identification of p2y9/GPR23 as a novel G protein-coupled receptor for lysophosphatidic acid, structurally distant from the Edg family. J. Biol. Chem..

[17] Benesch M.G., Ko Y.M., McMullen T.P., Brindley D.N. (2014). Autotaxin in the crosshairs: Taking aim at cancer and other inflammatory conditions. FEBS Lett..

[18] Watanabe N., Ikeda H., Nakamura K., Ohkawa R., Kume Y., Aoki J., Hama K., Okudaira S., Tanaka M., Tomiya T. (2007). Both plasma lysophosphatidic acid and serum autotaxin levels are increased in chronic hepatitis C. J. Clin. Gastroenterol..

[19] Nikitopoulou I., Oikonomou N., Karouzakis E., Sevastou I., Nikolaidou-Katsaridou N., Zhao Z., Mersinias V., Armaka M., Xu Y., Masu M. (2012). Autotaxin expression from synovial fibroblasts is essential for the pathogenesis of modeled arthritis. J. Exp. Med..

[20] Tokumura A., Carbone L.D., Yoshioka Y., Morishige J., Kikuchi M., Postlethwaite A., Watsky M.A. (2009). Elevated serum levels of arachidonoyl-lysophosphatidic acid and sphingosine 1-phosphate in systemic sclerosis. Int. J. Med. Sci..

[21] Park G.Y., Lee Y.G., Berdyshev E., Nyenhuis S., Du J., Fu P., Gorshkova I.A., Li Y., Chung S., Karpurapu M. (2013). Autotaxin production of lysophosphatidic acid mediates allergic asthmatic inflammation. Am. J. Respir. Crit. Care Med..

[22] Georas S.N., Berdyshev E., Hubbard W., Gorshkova I.A., Usatyuk P.V., Saatian B., Myers A.C., Williams M.A., Xiao H.Q., Liu M. (2007). Lysophosphatidic acid is detectable in human bronchoalveolar lavage fluids at baseline and increased after segmental allergen challenge. Clin. Exp. Allergy J. Br. Soc. Allergy Clin. Immunol..

[23] D’Souza K., Paramel G.V., Kienesberger P.C. (2018). Lysophosphatidic Acid Signaling in Obesity and Insulin Resistance. Nutrients.

[24] Khaw P.T., Migdal C.S. (1996). Current techniques in wound healing modulation in glaucoma surgery. Curr. Opin. Ophthalmol..

[25] Chiou A.G., Florakis G.J., Kazim M. (1998). Management of conjunctival cicatrizing diseases and severe ocular surface dysfunction. Surv. Ophthalmol..

[26] Chui J., Di Girolamo N., Wakefield D., Coroneo M.T. (2008). The pathogenesis of pterygium: Current concepts and their therapeutic implications. Ocul. Surf..

[27] Dale S.B., Saban D.R. (2015). Linking immune responses with fibrosis in allergic eye disease. Curr. Opin. Allergy Clin. Immunol..

[28] Munir S.Z., Aylward J. (2017). A Review of Ocular Graft-Versus-Host Disease. Optom. Vis. Sci..

[29] Broadway D.C., Chang L.P. (2001). Trabeculectomy, risk factors for failure and the preoperative state of the conjunctiva. J. Glaucoma.

[30] Schlunck G., Meyer-ter-Vehn T., Klink T., Grehn F. (2016). Conjunctival fibrosis following filtering glaucoma surgery. Exp. Eye Res..

[31] Hinz B. (2016). Myofibroblasts. Exp. Eye Res..

[32] Tomasek J.J., Gabbiani G., Hinz B., Chaponnier C., Brown R.A. (2002). Myofibroblasts and mechano-regulation of connective tissue remodelling. Nat. Rev. Mol. Cell Biol..

[33] Saika S., Yamanaka O., Okada Y., Tanaka S., Miyamoto T., Sumioka T., Kitano A., Shirai K., Ikeda K. (2009). TGF beta in fibroproliferative diseases in the eye. Front. Biosci. (Sch. Ed.).

[34] Cunliffe I.A., Richardson P.S., Rees R.C., Rennie I.G. (1995). Effect of TNF, IL-1, and IL-6 on the proliferation of human Tenon’s capsule fibroblasts in tissue culture. Br. J. Ophthalmol..

[35] Jampel H.D., Roche N., Stark W.J., Roberts A.B. (1990). Transforming growth factor-beta in human aqueous humor. Curr. Eye Res..

[36] Dan L., Chua C.K., Leong K.F. (2010). Fibroblast response to interstitial flow: A state-of-the-art review. Biotechnol. Bioeng..

[37] Gater R., Ipek T., Sadiq S., Nguyen D., Jones L., El Haj A., Yang Y. (2019). Investigation of Conjunctival Fibrosis Response Using a 3D Glaucoma Tenon’s Capsule + Conjunctival Model. Investig. Ophthalmol. Vis. Sci..

[38] Oouchi Y., Watanabe M., Ida Y., Ohguro H., Hikage F. (2021). Rosiglitasone and ROCK Inhibitors Modulate Fibrogenetic Changes in TGF-β2 Treated Human Conjunctival Fibroblasts (HconF) in Different Manners. Int. J. Mol. Sci..

[39] Cordeiro M.F., Chang L., Lim K.S., Daniels J.T., Pleass R.D., Siriwardena D., Khaw P.T. (2000). Modulating conjunctival wound healing. Eye.

[40] Cordeiro M.F., Occleston N.L., Khaw P.T. (1997). New concepts: Manipulation of the wound-healing response. Dev. Ophthalmol..

[41] O’Regan A., O’Brien C.J., Eivers S.B. (2021). The lysophosphatidic acid axis in fibrosis: Implications for glaucoma. Wound Repair Regen. Off. Publ. Wound Heal. Soc. Eur. Tissue Repair Soc..

[42] Wang X., Huai G., Wang H., Liu Y., Qi P., Shi W., Peng J., Yang H., Deng S., Wang Y. (2018). Mutual regulation of the Hippo/Wnt/LPA/TGF-β signaling pathways and their roles in glaucoma (Review). Int. J. Mol. Med..

[43] Honjo M., Igarashi N., Kurano M., Yatomi Y., Igarashi K., Kano K., Aoki J., Weinreb R.N., Aihara M. (2018). Autotaxin-Lysophosphatidic Acid Pathway in Intraocular Pressure Regulation and Glaucoma Subtypes. Investig. Ophthalmol. Vis. Sci..

[44] Watsky M.A., Griffith M., Wang D.A., Tigyi G.J. (2000). Phospholipid growth factors and corneal wound healing. Ann. N. Y. Acad. Sci..

[45] Igarashi N., Honjo M., Kurano M., Yatomi Y., Igarashi K., Kano K., Aoki J., Aihara M. (2018). Increased aqueous autotaxin and lysophosphatidic acid levels are potential prognostic factors after trabeculectomy in different types of glaucoma. Sci. Rep..

[46] Matsumura T., Fujimoto T., Futakuchi A., Takihara Y., Watanabe-Kitamura F., Takahashi E., Inoue-Mochita M., Tanihara H., Inoue T. (2020). TGF-β-induced activation of conjunctival fibroblasts is modulated by FGF-2 and substratum stiffness. PLoS ONE.

[47] Yin F., Watsky M.A. (2005). LPA and S1P increase corneal epithelial and endothelial cell transcellular resistance. Investig. Ophthalmol. Vis. Sci..

[48] Kaneko Y., Ohta M., Inoue T., Mizuno K., Isobe T., Tanabe S., Tanihara H. (2016). Effects of K-115 (Ripasudil), a novel ROCK inhibitor, on trabecular meshwork and Schlemm’s canal endothelial cells. Sci. Rep..

[49] Ichioka H., Hirohashi Y., Sato T., Furuhashi M., Watanabe M., Ida Y., Hikage F., Torigoe T., Ohguro H. (2023). G-Protein-Coupled Receptors Mediate Modulations of Cell Viability and Drug Sensitivity by Aberrantly Expressed Recoverin 3 within A549 Cells. Int. J. Mol. Sci..

[50] Hikage F., Atkins S., Kahana A., Smith T.J., Chun T.H. (2019). HIF2A-LOX Pathway Promotes Fibrotic Tissue Remodeling in Thyroid-Associated Orbitopathy. Endocrinology.

[51] Ota C., Ida Y., Ohguro H., Hikage F. (2020). ROCK inhibitors beneficially alter the spatial configuration of TGFβ2-treated 3D organoids from a human trabecular meshwork (HTM). Sci. Rep..

[52] Ida Y., Hikage F., Itoh K., Ida H., Ohguro H. (2020). Prostaglandin F2α agonist-induced suppression of 3T3-L1 cell adipogenesis affects spatial formation of extra-cellular matrix. Sci. Rep..

[53] Itoh K., Hikage F., Ida Y., Ohguro H. (2020). Prostaglandin F2α Agonists Negatively Modulate the Size of 3D Organoids from Primary Human Orbital Fibroblasts. Investig. Ophthalmol. Vis. Sci..

[54] Tsugeno Y., Furuhashi M., Sato T., Watanabe M., Umetsu A., Suzuki S., Ida Y., Hikage F., Ohguro H. (2022). FGF-2 enhances fibrogenetic changes in TGF-β2 treated human conjunctival fibroblasts. Sci. Rep..

[55] Tsugeno Y., Sato T., Watanabe M., Higashide M., Furuhashi M., Umetsu A., Suzuki S., Ida Y., Hikage F., Ohguro H. (2022). All Trans-Retinoic Acids Facilitate the Remodeling of 2D and 3D Cultured Human Conjunctival Fibroblasts. Bioengineering.

[56] Habanjar O., Diab-Assaf M., Caldefie-Chezet F., Delort L. (2021). 3D Cell Culture Systems: Tumor Application, Advantages, and Disadvantages. Int. J. Mol. Sci..

[57] Watanabe M., Sato T., Tsugeno Y., Higashide M., Furuhashi M., Umetsu A., Suzuki S., Ida Y., Hikage F., Ohguro H. (2022). An α2-Adrenergic Agonist, Brimonidine, Beneficially Affects the TGF-β2-Treated Cellular Properties in an In Vitro Culture Model. Bioengineering.

[58] van Meer G., Voelker D.R., Feigenson G.W. (2008). Membrane lipids: Where they are and how they behave. Nat. Rev. Mol. Cell Biol..

[59] Davenport A.P., Alexander S.P., Sharman J.L., Pawson A.J., Benson H.E., Monaghan A.E., Liew W.C., Mpamhanga C.P., Bonner T.I., Neubig R.R. (2013). International Union of Basic and Clinical Pharmacology. LXXXVIII. G protein-coupled receptor list: Recommendations for new pairings with cognate ligands. Pharmacol. Rev..

[60] Yung Y.C., Stoddard N.C., Chun J. (2014). LPA receptor signaling: Pharmacology, physiology, and pathophysiology. J. Lipid Res..

[61] Jeon E.S., Kim J.H., Ryu H., Kim E.K. (2012). Lysophosphatidic acid activates TGFBIp expression in human corneal fibroblasts through a TGF-β1-dependent pathway. Cell. Signal..

[62] Lim R. (2022). The surgical management of glaucoma: A review. Clin. Exp. Ophthalmol..

[63] Ohguro H., Ida Y., Hikage F., Umetsu A., Ichioka H., Watanabe M., Furuhashi M. (2022). STAT3 Is the Master Regulator for the Forming of 3D Spheroids of 3T3-L1 Preadipocytes. Cells.

[64] Yamanaka O., Kitano-Izutani A., Tomoyose K., Reinach P.S. (2015). Pathobiology of wound healing after glaucoma filtration surgery. BMC Ophthalmol..

[65] Zhang X., Li M., Yin N., Zhang J. (2021). The Expression Regulation and Biological Function of Autotaxin. Cells.

[66] Sumida H., Noguchi K., Kihara Y., Abe M., Yanagida K., Hamano F., Sato S., Tamaki K., Morishita Y., Kano M.R. (2010). LPA4 regulates blood and lymphatic vessel formation during mouse embryogenesis. Blood.

[67] Yukiura H., Hama K., Nakanaga K., Tanaka M., Asaoka Y., Okudaira S., Arima N., Inoue A., Hashimoto T., Arai H. (2011). Autotaxin regulates vascular development via multiple lysophosphatidic acid (LPA) receptors in zebrafish. J. Biol. Chem..

[68] Takara K., Eino D., Ando K., Yasuda D., Naito H., Tsukada Y., Iba T., Wakabayashi T., Muramatsu F., Kidoya H. (2017). Lysophosphatidic Acid Receptor 4 Activation Augments Drug Delivery in Tumors by Tightening Endothelial Cell-Cell Contact. Cell Rep..

[69] Eino D., Tsukada Y., Naito H., Kanemura Y., Iba T., Wakabayashi T., Muramatsu F., Kidoya H., Arita H., Kagawa N. (2018). LPA4-Mediated Vascular Network Formation Increases the Efficacy of Anti-PD-1 Therapy against Brain Tumors. Cancer Res..

[70] Hata E., Sasaki N., Takeda A., Tohya K., Umemoto E., Akahoshi N., Ishii S., Bando K., Abe T., Kano K. (2016). Lysophosphatidic acid receptors LPA4 and LPA6 differentially promote lymphocyte transmigration across high endothelial venules in lymph nodes. Int. Immunol..

[71] Igarashi H., Akahoshi N., Ohto-Nakanishi T., Yasuda D., Ishii S. (2015). The lysophosphatidic acid receptor LPA4 regulates hematopoiesis-supporting activity of bone marrow stromal cells. Sci. Rep..

[72] Liliom K., Guan Z., Tseng J.L., Desiderio D.M., Tigyi G., Watsky M.A. (1998). Growth factor-like phospholipids generated after corneal injury. Am. J. Physiol..

[73] Watanabe M., Ida Y., Ohguro H., Ota C., Hikage F. (2021). Establishment of appropriate glaucoma models using dexamethasone or TGFβ2 treated three-dimension (3D) cultured human trabecular meshwork (HTM) cells. Sci. Rep..

[74] Katayama H., Furuhashi M., Umetsu A., Hikage F., Watanabe M., Ohguro H., Ida Y. (2021). Modulation of the Physical Properties of 3D Spheroids Derived from Human Scleral Stroma Fibroblasts (HSSFs) with Different Axial Lengths Obtained from Surgical Patients. Curr. Issues Mol. Biol..

[75] Ida Y., Umetsu A., Furuhashi M., Watanabe M., Tsugeno Y., Suzuki S., Hikage F., Ohguro H. (2022). ROCK 1 and 2 affect the spatial architecture of 3D spheroids derived from human corneal stromal fibroblasts in different manners. Sci. Rep..

[76] Ohguro H., Watanabe M., Sato T., Hikage F., Furuhashi M., Okura M., Hida T., Uhara H. (2023). 3D Spheroid Configurations Are Possible Indictors for Evaluating the Pathophysiology of Melanoma Cell Lines. Cells.

[77] Miyamoto S., Nishikiori N., Sato T., Watanabe M., Umetsu A., Tsugeno Y., Hikage F., Sasaya T., Kato H., Ogi K. (2023). Three-Dimensional Spheroid Configurations and Cellular Metabolic Properties of Oral Squamous Carcinomas Are Possible Pharmacological and Pathological Indicators. Cancers.

