# Supplementary material for: Lysophosphatidic Acid Modulates TGF-β2-Induced Biological Phenotype in Human Conjunctival Fibroblasts
Source: Life (Basel). 2024 Jun 17;14(6):770. doi: 10.3390/life14060770 (PMC11204428; doi:10.3390/life14060770)
Supplement: Supplementary file 1 [file life-14-00770-s001.zip › life-3004273-supplementary.pdf]

**Supplemental Fig. 1 The mRNA expression of LPAR 1-6 of 2D cultured HconF cells.**

2D HconF cells were subjected to qPCR analysis to estimate the expression of mRNA in *LPAR 1-6*. All experiments were performed in duplicate using 3 different confluent 6-well dishes (2D). *$P$<0.05, **$P$<0.01, ***$P$<0.005.

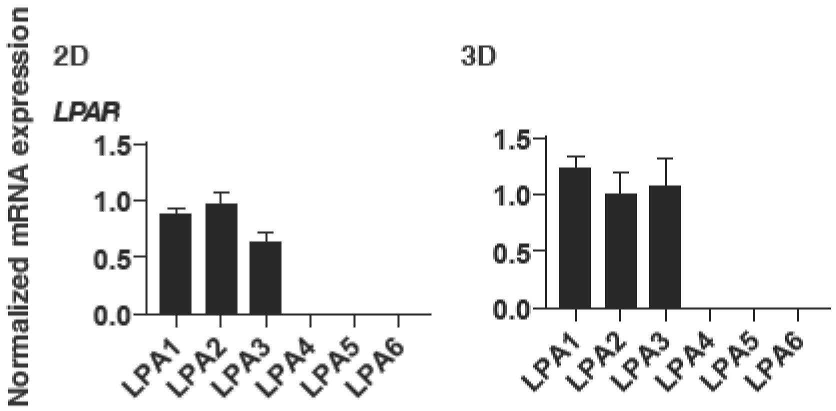


**Supplemental Fig. 2 Immunolabeling of ECMs of the 2D Cultured HconF cells.**

2D HconF cells were untreated or treated with a 5 ng/ml solution of TGF-β2 in the absence or presence of 500 nM LPA., At Day 6, each sample was subjected to immunostaining for *COL 1*, *COL 4*, *COL 6*, *FN* and *a-SMA*. All experiments were performed in duplicate using fresh preparations (n=5).

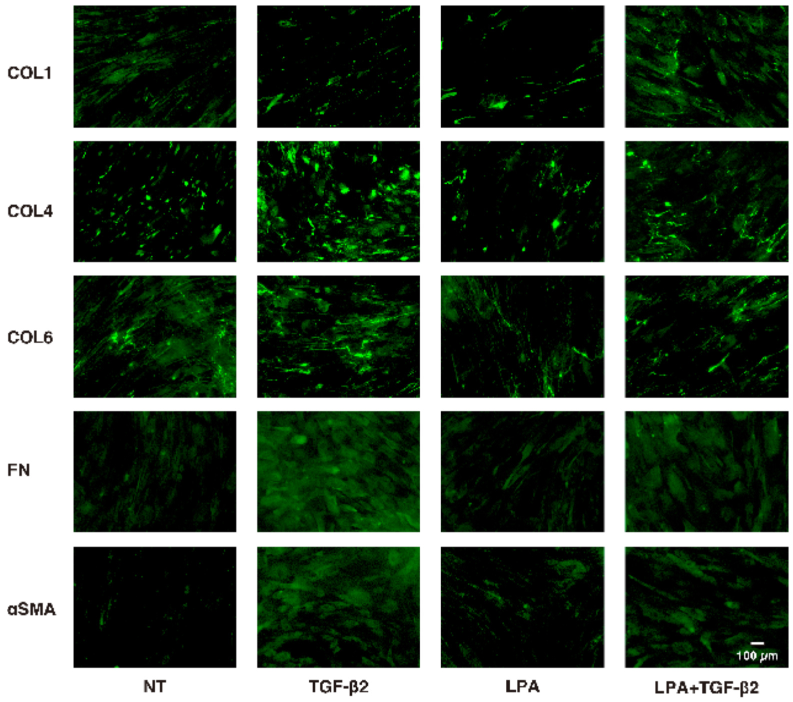

**Supplemental Table 1. The sequences of primers and probes used in this study.**

| | | Sequence | Exon Location | RefSeq Number |
|---|---|---|---|---|
| human RPLP0 | Probe | 5'-/56-FAM/CCCTGTCTT/ZEN/CCCTGGGCATCAC/3IABkFQ/-3' | 2-3 | NM_001002 |
| | Primer2 | 5'-TCGTCTTTAAACCCTGCGTG-3' | | |
| | Primer1 | 5'-TGTCTGCTCCCACAATGAAAC-3' | | |
| human COL1A1 | Probe | 5'-/56-FAM/TCGAGGGCC/ZEN/AAGACGAAGACATC/3IABkFQ/-3' | 1-2 | NM_000088 |
| | Primer2 | 5'-GACATGTTCAGCTTTGTGGAC-3' | | |
| | Primer1 | 5'-TTCTGTACGCAGGTGATTGG-3' | | |
| human COL4A1 | Probe | 5′-/56-FAM/TCATACAGA/ZEN/CTTGGCAGCGGCT/3IABkFQ/-3′ | 51-52 | NM_001845 |
| | Primer2 | 5′-AGAGAGGAGCGAGATGTTCA-3′ | | |
| | Primer1 | 5′-TGAGTCAGGCTTCATTATGTTCT-3′ | | |
| human COL6A1 | Primer2 | 5'-CCTCGTGGACAAAGTCAAGT-3' | 2-3 | NM_001848 |
| | Primer1 | 5'-GTGAGGCCTTGGATGATCTC-3' | | |
| human FN1 | Primer2 | 5'-CGTCCTAAAGACTCCATGATCTG-3' | 3-4 | NM_212482 |
| | Primer1 | 5'-ACCAATCTTGTAGGACTGACC-3' | | |
| human $\alpha$SMA | Probe | 5'-/56-FAM/AGACCCTGT/ZEN/TCCAGCCATCCTTC/3IABkFQ/-3' | 8-9 | NM_001613 |
| | Primer2 | 5'-AGAGTTACGAGTTGCCTGATG-3' | | |
| | Primer1 | 5'-CTGTTGTAGGTGGTTTCATGGA-3' | | |
| human TIMP1 | Probe | 5′-/56-FAM/TCAACCAGA/ZEN/CCACCTTATACCAGCG/3IABkFQ/-3′ | 2-4 | NM_003254 |
| | Primer2 | 5′-CCTTCTGCAATTCCGACCT-3′ | | |
| | Primer1 | 5′-GCTTGGAACCCTTTATACATCTTG-3′ | | |
| human TIMP2 | Probe | 5′-/56-FAM/TCTCATTGC/ZEN/AGGAAAGGCCGAGG/3IABkFQ/-3′ | 3-4 | NM_003255 |
| | Primer2 | 5′-GACGTTGGAGGAAAGAAGGA-3′ | | |
| | Primer1 | 5′-TGTGGTTCAGGCTCTTCTTC-3′ | | |
| human TIMP3 | Probe | 5′-/56-FAM/CCTCCTTTA/ZEN/CCAGCTTCTTCCCCAC/3IABkFQ/-3′ | 1-3 | NM_000362 |
| | Primer2 | 5′-CCTTCTGCAACTCCGACATC-3′ | | |

| | | | | |
|---|---|---|---|---|
| | Primer1 | 5′-CGGTACATCTTCATCTGCTTGA-3′ | | |
| human TIMP4 | Probe | 5′-/56-FAM/ACTGAGGAC/ZEN/CTGACCAGTCAAGAGA/3IABkFQ/-3′ | 3-4 | NM_003256 |
| | Primer2 | 5′-GGTTTGAGAAAGTCAAGGATGTTC-3′ | | |
| | Primer1 | 5′-GTTGCACAGATGGATGAAGAC-3′ | | |
| human MMP2 | Primer2 | 5′-TCCACCACCTACAACTTTGAG-3′ | 6-7 | NM_004530 |
| | Primer1 | 5′-GTGCAGCTGTCATAGGATGT-3′ | | |
| human MMP9 | Primer2 | 5′-ACATCGTCATCCAGTTTGGTG-3′ | 3-4 | NM_004994 |
| | Primer1 | 5′-CGTCGAAATGGGCGTCT-3′ | | |
| human MMP14 | Primer2 | 5′-TTCGCCGACTAAGCAGAAG-3′ | 1-1 | NM_004995 |
| | Primer1 | 5′-CTTGAATTCCTAGACCGCTGT-3′ | | |
| human LPAR1 | Primer2 | 5’-ACAGTGATTCCAAGTCCCATC-3’ | 3-4 | NM_057159 |
| | Primer1 | 5’-GTAATTTCACAGCCCCAGTTC-3’ | | |
| human LPAR2 | Primer2 | 5’-GTACTTTTCTACAGCCAGGACA-3’ | 2-3 | NM_004720 |
| | Primer1 | 5’-AGCCTGGTCAAGACTGTTG-3’ | | |
| human LPAR3 | Primer2 | 5’-AGCAGCAGGAACCACCT-3’ | 2-3 | NM_012152 |
| | Primer1 | 5’-ACCCATGAAGCTAATGAAGACG-3’ | | |
| human LPAR4 | Primer2 | 5’-GGTTCACCACTCTGACACTATG-3’ | 1-2 | NM_005296 |
| | Primer1 | 5’-TCTACAGGCATCAGCACATTC-3’ | | |
| human LPAR5 | Primer2 | 5’-GTCATGGGAATGTGGGCTAT-3’ | 1-3 | NM_020400 |
| | Primer1 | 5’-AGAGCAACACGGAGCAC-3’ | | |
| human LPAR6 | Primer2 | 5’-CCTCCAGCAAATTCCAGCA-3’ | 4-5 | NM_005767 |
| | Primer1 | 5’-GGTACAATCAAAGCTCACTGC-3’ | | |
